# Adalimumab-Associated Transverse Myelitis Revealing Relapsing-Remitting Multiple Sclerosis in a Patient With Crohn’s Disease: A Case Report

**DOI:** 10.7759/cureus.97651

**Published:** 2025-11-24

**Authors:** Sara S Gawargeous, Taha Elsahy

**Affiliations:** 1 Acute Medicine, Peterborough City Hospital, Peterborough, GBR

**Keywords:** adalimumab, crohn’s disease, demyelination, multiple sclerosis, oligoclonal bands, tnf-alpha inhibitor, transverse myelitis

## Abstract

We report the case of a 40-year-old woman with a background of Crohn’s disease treated with azathioprine and adalimumab, who presented with acute right leg weakness, sensory loss, and urinary hesitancy. The MRI revealed a longitudinal cervical spinal cord lesion consistent with transverse myelitis (TM) and a supratentorial white matter lesion. The CSF analysis demonstrated unmatched oligoclonal bands, supporting central nervous system (CNS) demyelination. A prior episode of facial numbness in 2022 was retrospectively recognized as a previous relapse. These findings fulfilled the McDonald criteria for relapsing-remitting multiple sclerosis (RRMS). This case highlights the diagnostic challenges in patients with autoimmune disease receiving anti-tumor necrosis factor (TNF) therapy and underscores the importance of considering multiple sclerosis (MS) in such clinical contexts. The patient was treated with corticosteroids, while adalimumab was discontinued, and she demonstrated gradual neurological improvement over several weeks.

## Introduction

Multiple sclerosis (MS) is a chronic, immune-mediated demyelinating disorder of the central nervous system (CNS). Transverse myelitis (TM) is a rare but recognized initial manifestation of MS. Multiple sclerosis commonly presents with motor or sensory deficits, is diagnosed through MRI and CSF oligoclonal bands, and is typically treated with corticosteroids acutely and disease-modifying therapy long-term. Diagnosis becomes more complex when overlapping autoimmune diseases such as Crohn’s disease are present, especially in patients receiving immunosuppressive or biologic agents such as anti-tumor necrosis factor-alpha (TNF-α) therapies. Adalimumab is a fully human monoclonal antibody against TNF-α, widely used for autoimmune diseases like rheumatoid arthritis, ankylosing spondylitis, and Crohn’s disease. Although effective, it has been associated with rare but serious adverse events, including CNS demyelination [1-3].

## Case presentation

A 40-year-old woman with a 10-year history of Crohn’s disease, treated with azathioprine (200 mg daily) and adalimumab, presented with a three-day history of progressive right lower limb numbness and weakness. Initial symptoms included tingling in the right upper abdominal quadrant, progressing to right leg heaviness and weakness, and urinary hesitancy. She denied bowel incontinence, trauma, infection, or visual changes.

Examination

On examination, she was alert and oriented with a Glasgow coma scale (GCS) score of 15. Cranial nerve examination was normal. Power and sensation were intact in both upper limbs. In the lower limbs, power was reduced in the right leg (grade 1-2/5) with impaired pinprick, pain, and temperature sensation. The left leg had full strength (5/5). Reflexes were brisk bilaterally, and the right plantar response was extensor. The sensory level was variably reported around T4 to T6.

Investigations

**Figure 1:**
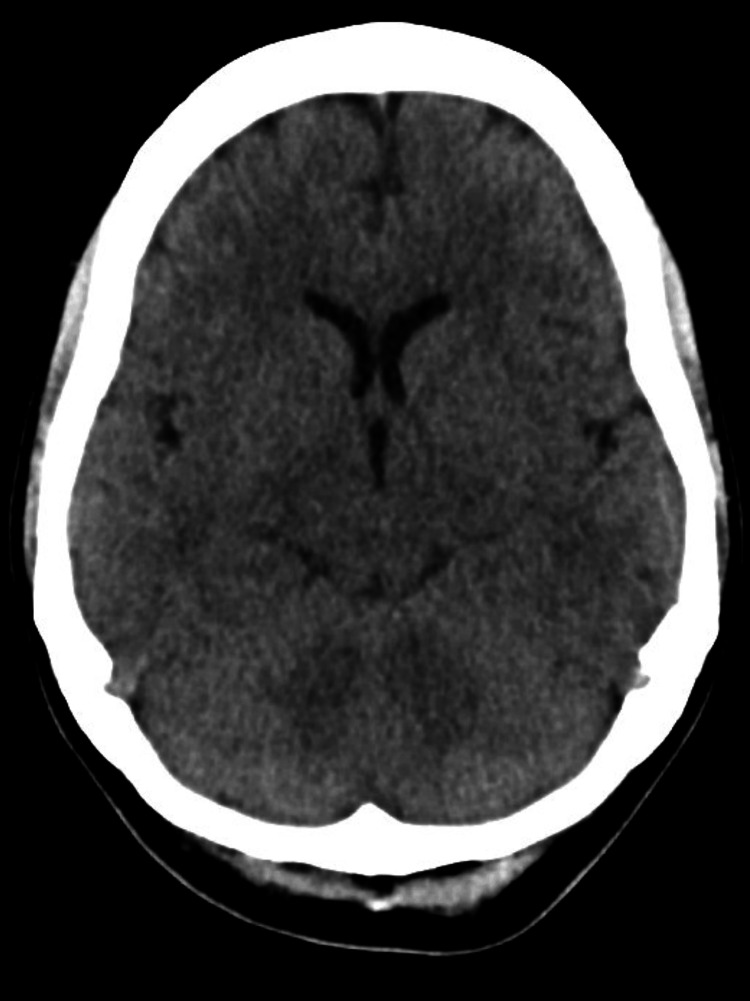
Normal axial CT brain showing no acute intracranial abnormality Axial non-contrast CT scan of the brain showing preserved gray-white differentiation and no evidence of hemorrhage, infarction, or mass effect.

**Figure 2:**
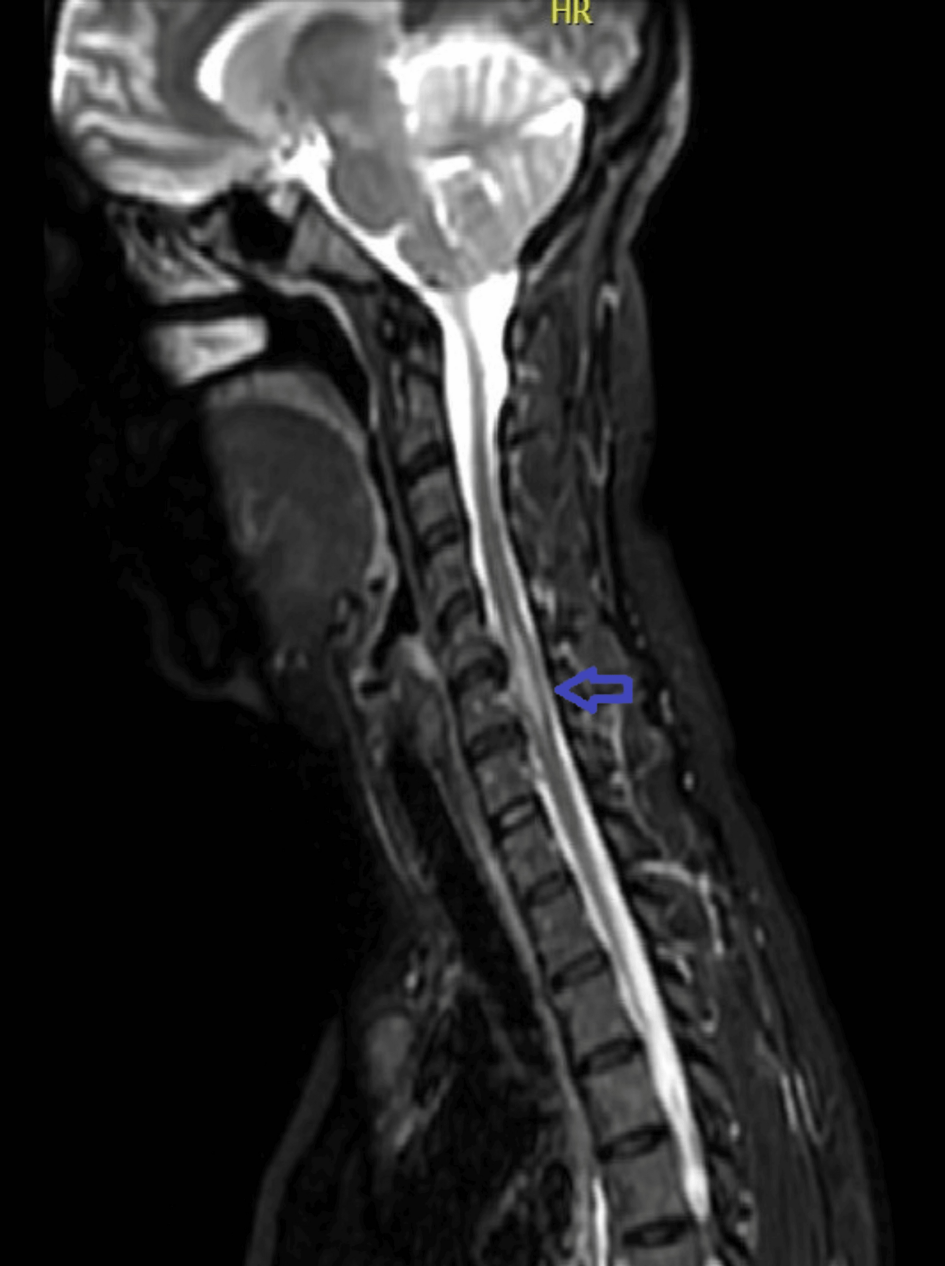
Cervical spine MRI showing TM Sagittal T2-weighted MRI of the cervical spine showing a hyperintense intramedullary lesion extending from C5 to C7 (arrow), with mild cord expansion and no contrast enhancement, consistent with TM. TM: Transverse myelitis

**Figure 3:**
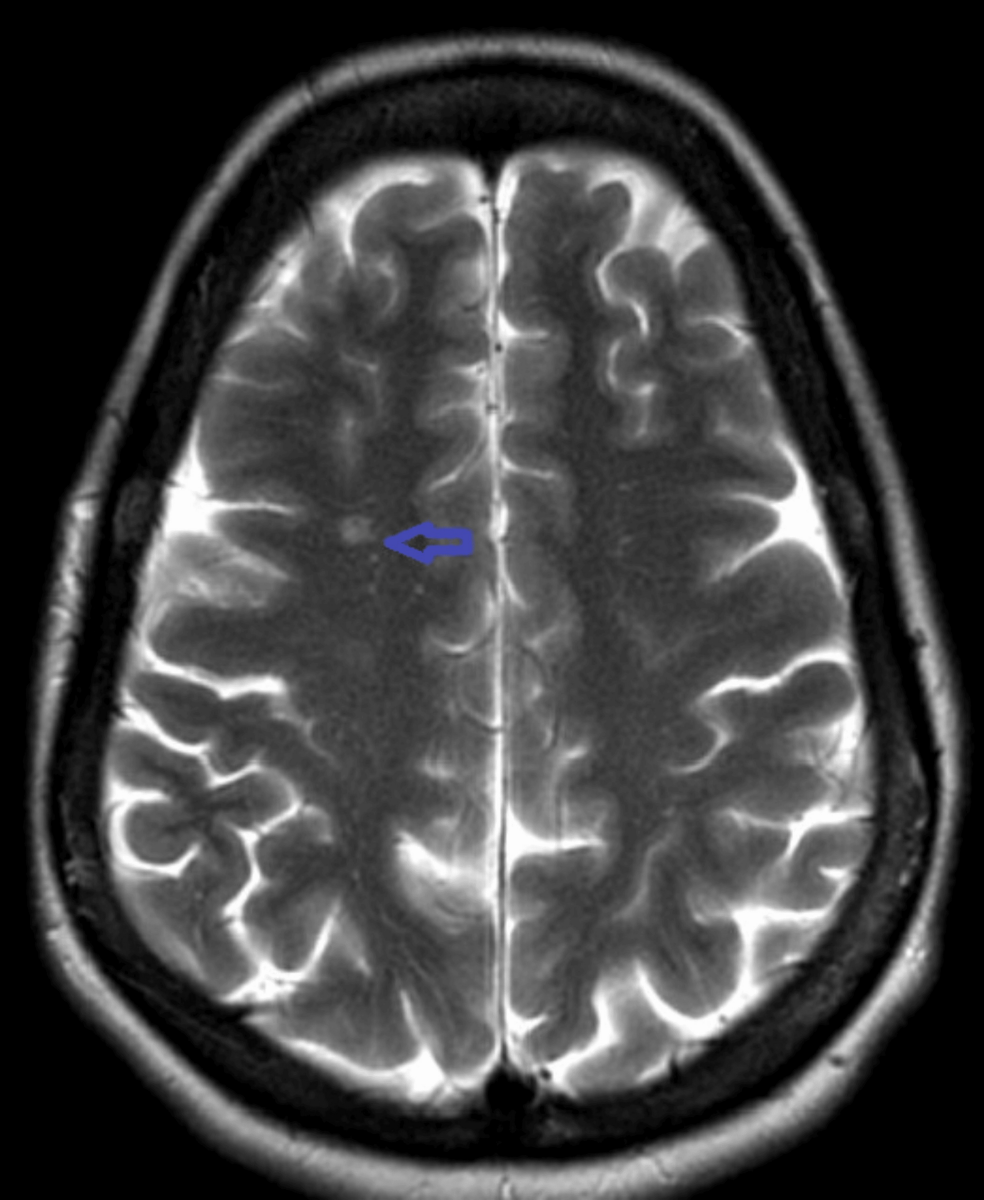
Axial brain MRI demonstrating a demyelinating lesion Axial T2-weighted MRI of the brain showing a small hyperintense subcortical white-matter lesion (arrow) consistent with demyelination.

A CT scan of the head was unremarkable (Figure 1). The MRI of the spine revealed a T2 hyperintense intramedullary lesion extending from C5 to C7, with mild cord expansion and no contrast enhancement, consistent with TM (Figure 2). Additionally, a new ovoid subcortical white matter lesion was identified on brain MRI, suggestive of a possible demyelinating process (Figure 3).

Serum testing for autoimmune and inflammatory markers, including antinuclear antibodies (ANA), antineutrophil cytoplasmic antibodies (ANCA), aquaporin-4 (AQP4) antibodies, and myelin oligodendrocyte glycoprotein (MOG) antibodies, was negative. Screening for hepatitis B and C, HIV, vitamin B12, folate, thyroid function, and HbA1c were all within normal limits. The patient’s blood and biochemical investigations are summarized in Table 1. Given her previous bariatric surgery, serum folate and vitamin B12 levels were checked to exclude subacute combined degeneration of the spinal cord, and both were within normal limits.

**Table 1:** Blood and biochemical investigations Routine blood, liver, renal, and inflammatory markers were within normal limits. Autoimmune screening was negative. ANA:* *Antinuclear antibody

Test	Result	Reference range (units)
Hemoglobin	131 g/L	115–165
White cell count	7.2 ×10⁹/L	4.0–11.0
Platelets	250 ×10⁹/L	150–400
Neutrophils	4.2 ×10⁹/L	1.8–7.7
Lymphocytes	2.0 ×10⁹/L	1.4–4.8
Aspartate transaminase (AST)	14 IU/L	10–35
Alanine transaminase (ALT)	6 IU/L	<33
Alkaline phosphatase (ALP)	51 IU/L	30–130
Albumin	41 g/L	35–50
Total bilirubin	8 µmol/L	0–21
Total protein	63 g/L	60–80
Globulin	22 g/L	20-35
Sodium	135 mmol/L	133–146
Potassium	4.4 mmol/L	3.5–5.3
Chloride	106 mmol/L	95–108
Urea	2.6 mmol/L	2.5–7.8
Creatinine	68 µmol/L	45–84
Estimated glomerular filtration rate (eGFR)	>90 mL/min/1.73m²	>60
Erythrocyte sedimentation rate (ESR)	2 mm/h	0–15
C-reactive protein (CRP)	1 mg/L	<5
ANA	0.2 (Negative)	<0.7 (Negative)
Anti-dsDNA, ENA (Ro, La, Sm, RNP, Jo-1, Scl-70, etc.)	Negative	Negative

The CSF analysis was acellular, with normal glucose and mildly elevated protein levels. Oligoclonal bands were positive in the CSF but not in serum, indicating intrathecal IgG synthesis. Viral polymerase chain reaction (PCR) for HSV-1/2, varicella-zoster virus (VZV), enterovirus, and parechovirus were all negative. Detailed CSF findings are presented in Table 2.

**Table 2:** The CSF analysis The CSF findings demonstrated unmatched oligoclonal bands consistent with intrathecal IgG synthesis, while viral PCRs were negative. PCRs: Polymerase chain reactions

Test	Result	Reference range (units)
Appearance	Clear, colourless	Clear
White cells	<2 ×10⁶/L	<5 ×10⁶/L
Red cells	None	<5 ×10⁶/L
Protein	0.37 g/L	0.15–0.35
Glucose	2.2 mmol/L	2.2–4.0
Oligoclonal bands	Positive (≥10 IgG bands)	Negative
Paired serum oligoclonal bands	Negative	Negative
Viral PCR (HSV-1, HSV-2, VZV, enterovirus, parechovirus)	Not detected	Negative

Management and hospital course

The patient was initially evaluated for a spinal cord stroke versus an inflammatory myelopathy. The head CT was unremarkable. Based on MRI findings and neurology input, she was treated with oral methylprednisolone 500 mg once daily for five days. She demonstrated slow but steady improvement and began rehabilitation with physiotherapy. She regained some motor function in the affected leg and was discharged with outpatient neurology follow-up. Azathioprine was continued, while adalimumab was held due to concerns about TNF-α inhibitor-associated neuroinflammation.

Follow-up and diagnosis

In the outpatient neurology clinic, a diagnosis of relapsing-remitting multiple sclerosis (RRMS) was made based on clinical presentation, CSF findings, and MRI evidence of dissemination in time and space, as defined by the McDonald criteria. A prior self-limiting episode of facial numbness in 2022 was retrospectively considered a probable relapse, further supporting the diagnosis. The patient was referred to the MS service and commenced on appropriate disease-modifying therapy.

## Discussion

This case highlights the diagnostic challenge of distinguishing MS from TNF-α inhibitor-associated demyelination in patients with autoimmune disease. Transverse myelitis may represent the first presentation of MS, particularly in the setting of a compatible history and supportive CSF findings. Anti-TNF-α therapies such as adalimumab have been increasingly linked to CNS demyelination, including MS, optic neuritis, and TM [1-7]. A systematic review by Sicotte and Voskuhl identified over 120 cases of CNS demyelination in patients receiving anti-TNF agents [4]. Case reports have described similar presentations in Crohn’s disease patients on adalimumab developing longitudinally extensive TM or brain demyelinating lesions [1,5,7].

Several reports have documented CNS demyelination in association with anti-TNF-α therapies, including adalimumab. Kaltsonoudis et al. reviewed numerous such cases, with MS being the most frequent manifestation, followed by optic neuritis and TM [7]. In their analysis, most events occurred within two years of initiating anti-TNF-α therapy and often improved following drug discontinuation. Case reports have similarly described Crohn’s disease patients developing longitudinally extensive TM while on adalimumab, supporting the possibility that TNF-α blockade may unmask or precipitate demyelinating disease in susceptible individuals [1,3,8].

The underlying mechanism remains uncertain but is thought to involve immune dysregulation secondary to TNF-α inhibition, leading to a shift from peripheral to central inflammatory activity [5,7]. While causality cannot always be proven, the temporal relationship between adalimumab exposure and symptom onset, along with improvement following cessation, strengthens the argument for drug-induced demyelination [7,9]. Our case aligns with previously published reports and emphasizes the importance of considering demyelination in any patient presenting with new neurological symptoms while receiving anti-TNF therapy [10]. Prompt recognition and withdrawal of the offending agent are crucial to prevent irreversible neurological damage.

## Conclusions

Transverse myelitis can be the initial manifestation of RRMS, especially in patients with autoimmune diseases like Crohn’s. In the context of biologic therapy, clinicians should maintain a high index of suspicion for demyelinating disease. Early imaging and CSF analysis are crucial for accurate diagnosis and timely initiation of MS-specific therapy. Distinguishing drug-induced demyelination from unmasking MS influences long-term management and selection of disease-modifying therapy. This case highlights the importance of recognizing possible demyelination in patients treated with anti-TNF therapy and modifying treatment promptly.
